# Characterization of an anti-fetal AChR monoclonal antibody isolated from a myasthenia gravis patient

**DOI:** 10.1038/s41598-017-14350-8

**Published:** 2017-10-31

**Authors:** Abhishek Saxena, Jo Stevens, Hakan Cetin, Inga Koneczny, Richard Webster, Konstantinos Lazaridis, Socrates Tzartos, Kathleen Vrolix, Gisela Nogales-Gadea, Barbie Machiels, Peter C. Molenaar, Jan Damoiseaux, Marc H. De Baets, Katja Simon-Keller, Alexander Marx, Angela Vincent, Mario Losen, Pilar Martinez-Martinez

**Affiliations:** 1 Department of Psychiatry and Neuropsychology, School for Mental Health and Neuroscience, Maastricht University, Maastricht, Netherlands; 2 Nuffield Department of Clinical Neurosciences, John Radcliffe University Hospital, Oxford, UK; 3 Hellenic Pasteur Institute, 127 Vasilissis Sofias Avenue 115 21, Ampelokipi, Athens, Greece; 4 Central Diagnostic Laboratory, Maastricht University Medical Center, Maastricht, Netherlands; 5 Institute of Pathology, University Medical Centre Mannheim, University of Heidelberg, Theodor-Kutzer-Ufer 1-3, 68167 Mannheim, Germany; 6 Present Address: Laboratory of Antibody Engineering, Shanghai Institute for Advanced Immunochemical Studies, ShanghaiTech University, Shanghai, China; 7 Present Address: Neuromuscular and Neuropediatric Diseases Research Group, Institut d’Investigació en Ciències de la Salut Germans Trias i Pujol i Campus Can Ruti, Universitat Autònoma de Barcelona, Badalona, Spain

## Abstract

We report here the sequence and functional characterization of a recombinantly expressed autoantibody (mAb 131) previously isolated from a myasthenia gravis patient by immortalization of thymic B cells using Epstein-Barr virus and TLR9 activation. The antibody is characterized by a high degree of somatic mutations as well as a 6 amino acid insertion within the VHCDR2. The recombinant mAb 131 is specific for the γ-subunit of the fetal AChR to which it bound with sub-nanomolar apparent affinity, and detected the presence of fetal AChR on a number of rhabdomyosarcoma cell lines. Mab 131 blocked one of the two α-bungarotoxin binding sites on the fetal AChR, and partially blocked the binding of an antibody (mAb 637) to the α-subunit of the AChR, suggesting that both antibodies bind at or near one ACh binding site at the α/γ subunit interface. However, mAb 131 did not reduce fetal AChR ion channel currents in electrophysiological experiments. These results indicate that mAb 131, although generated from an MG patient, is unlikely to be pathogenic and may make it a potentially useful reagent for studies of myasthenia gravis, rhabdomyosarcoma and arthrogryposis multiplex congenita which can be caused by fetal-specific AChR-blocking autoantibodies.

## Introduction

Myasthenia gravis (MG) with antibodies against the acetylcholine receptor (AChR) is an autoimmune disease of the neuromuscular junction (NMJ)^1–3^. The anti-AChR-autoantibodies cause loss of AChRs from the postsynaptic muscle membrane of the NMJ, resulting in failure of neuromuscular transmission and muscle weakness. The AChR is a ligand-gated ion channel and exists in fetal and adult forms each comprising four homologous subunits. Two α1, one β1 and one δ-subunit are expressed in combination with either the γ (“fetal” form, predominantly in developing muscle fibers) or the ε-subunit (“adult” form, at the NMJ in innervated muscle fibers). The AChR opens upon binding of acetylcholine (ACh) molecules to sites located on the α/ε (or α/γ) and α/δ interfaces. Antibodies against both the fetal (α_2_βγδ) and the adult (α_2_βεδ) AChR are found in MG patients, many binding to the shared α-subunits, but some patients have additional antibodies specific for the fetal γ-subunit, or more rarely the adult ε-subunit^4–7^.

AChR autoantibodies are predominantly IgG1 with some IgG3, and little IgG4. They lead to a decrease of AChR numbers and/or loss of function by three mechanisms^8^: antigenic modulation, in which antibodies divalently cross-link adjacent receptors leading to internalization and degradation of antibody/antigen complexes; complement-dependent damage caused by IgG1 and IgG3 subclass antibodies^9,10^; and less commonly functional inhibition of the ACh binding site so that the AChR channel does not open in response to released ACh^8^. During human development, the fetal AChR is expressed at the NMJ, and only replaced by the adult form from about 30 weeks gestation. In rare cases fetal-specific antibodies in a mother (with or without adult AChR antibodies and clinical MG) cross the placenta and inhibit the function of the fetal AChR leading to arthrogryposis multiplex congenita (AMC), a condition resulting from lack of fetal movement. AMC comprises multiple joint contractures, lung hypoplasia and profound respiratory impairment with the risk of fetal death or neonatal mortality^7,11–14^.

The fetal AChR (α_2_βγδ) is also expressed in human rhabdomyosarcoma (RMS) cells, which is the most common type of soft tissue malignancy in children^15^. Most patients with RMS respond well to available standard treatments including chemotherapy and irradiation, with 5-year survival rates of >70%^16^. In an experimental model, RMS cells proved sensitive to treatment with an immunotoxin-labeled human anti-γ-AChR scFv antibody fragment; possibly this finding opens a new approach for efficient, highly specific treatment^17^.

Monoclonal/recombinant (auto)antibodies against the AChR are essential tools for the study of MG, AMC and RMS. One suitable source for cloning AChR antibodies are the B cells derived from the thymus removed from patients with MG^18–21^.

In a previous study using the thymus of an early-onset MG patient, we generated a monoclonal B-cell line against the γ-subunit of the fetal AChR (clone P90-131)^20^. The monoclonal antibody (IgG1 isotype, mAb 131) did not cross-react with AChRs of mouse, rat or rhesus monkey muscle which are of the adult type and also did not bind to AChR from *Torpedo* electric organ which is of the gamma type (independent of development). In view of the possible application in RMS, we characterized the cloned anti-γ-AChR antibody in more detail. We used the B-cell receptor DNA sequence of clone P90-131 to produce the corresponding recombinant IgG1-131 and IgG4-131 antibodies, in order to determine their properties in relation to AChR-MG, AMC and RMS.

## Results

### Analysis of the mAb 131 sequence

**Figure 1:**
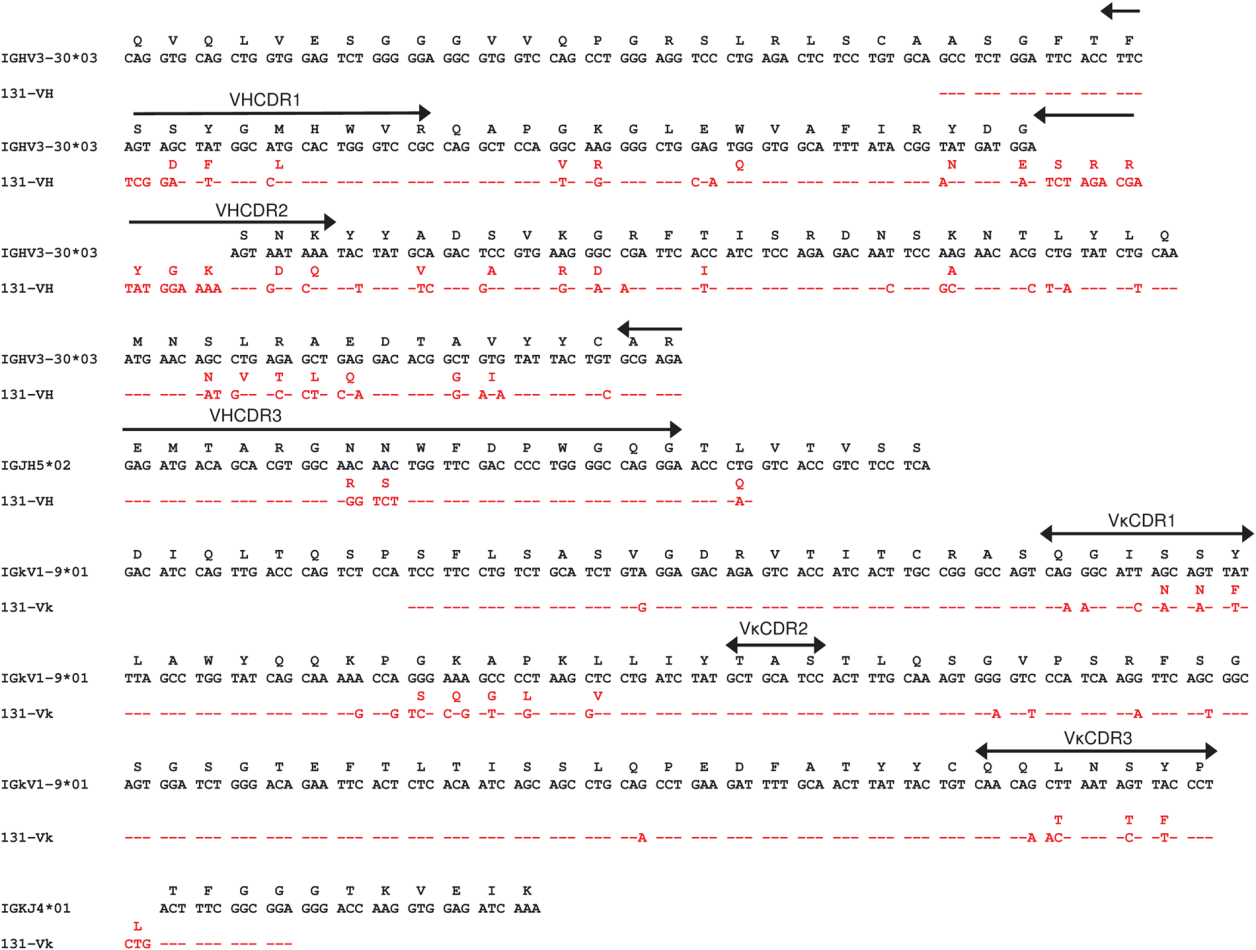
Sequence of the 131 VH and Vκ genes. IgG1**-**131 VH and Vκ nucleotide/amino acid sequences (red) were analyzed using IMGT/V-QUEST program and aligned with the closest germline alleles (black). A hyphen represents nucleotide identity with the germline sequence. VH and Vκ CDRs are marked within arrowed lines.

The complete sequences of the VH and Vκ chains of mAb 131 annotated with the complementarity-determining regions (CDR)s are shown in Fig. 1. The replacement to silent (R/S) mutation ratio, amino acid changes and mutational hotspot analysis of V gene segments of mAb 131 are summarized in Tables 1 and 2 respectively. The VH gene segment of mAb 131 differentiated from the V3-30*03 (L26401) and IGHJ5*02 germline genes as suggested by 55.7% and 82.9% homology, respectively. The sequence encoding the CDR 1, 2 and the framework regions (FR) of the VH gene had a high replacement to silent (R/S) mutation ratio. There were a large number of mutations in the RGYW and WRCY hotspot region of the VH including an 18 bp (6 AA) insertion in the CDR2. The Vκ and Jκ sequence shared 89.1% and 92.9% homology to V1-9*01 (K02096) and IGKJ4*01 alleles, respectively, with multiple mutations observed in VκCDR1 and VκCDR3 while the VκCDR2 used the conserved sequence from V1-9*01. Comparing the IgV gene of mAb 131 to the IgV genes of previously described anti-AChR antibodies^7,20,22–25^, no V gene was preferentially/ dominantly used between the different antibodies.

**Table 1:** Analysis of R/S ratio and amino acid changes in V gene segments of mAb 131.

	VH	VHFR1	VHCDR1	VHFR2	VHCDR2	VHFR3	VHCDR3^*^
R/S nt	91/9	8/0	17/0	33/2	11/0	22/7	0/0
AA Change/Identical	48/30	5/0	7/1	15/2	7/1	14/24	0/2
	**Vκ**	**VκFR1**	**VκCDR1**	**VκFR2**	**VκCDR2**	**VκFR3**	**VκCDR3**
R/S nt	16/13	0/1	4/2	7/3	1/0	0/5	4/2
AA Change/Identical	13/74	0/18	4/2	5/12	1/2	0/36	3/4

*IMGT/V-QUEST V gene analysis excludes DH and JH regions.

**Table 2:** Analysis of rgwy and wrcy mutational hotspots in V gene segments of mAb 131.

	FR1	CDR1	FR2	CDR2	FR3	CDR3
VH rgwy	2	1	3	1	1	0
VH wrcy	3	1	1	0	5	0
Vκ rgwy	1	2	3	0	3	2
Vκ wrcy	1	0	5	1	4	2

*Source - IMGT/V-QUEST programm (* http://www.imgt.org/IMGT_vquest/share/textes/).

### Production and validation of recombinant human IgG1 and IgG4 isotypes of mAb 131

**Figure 2:**
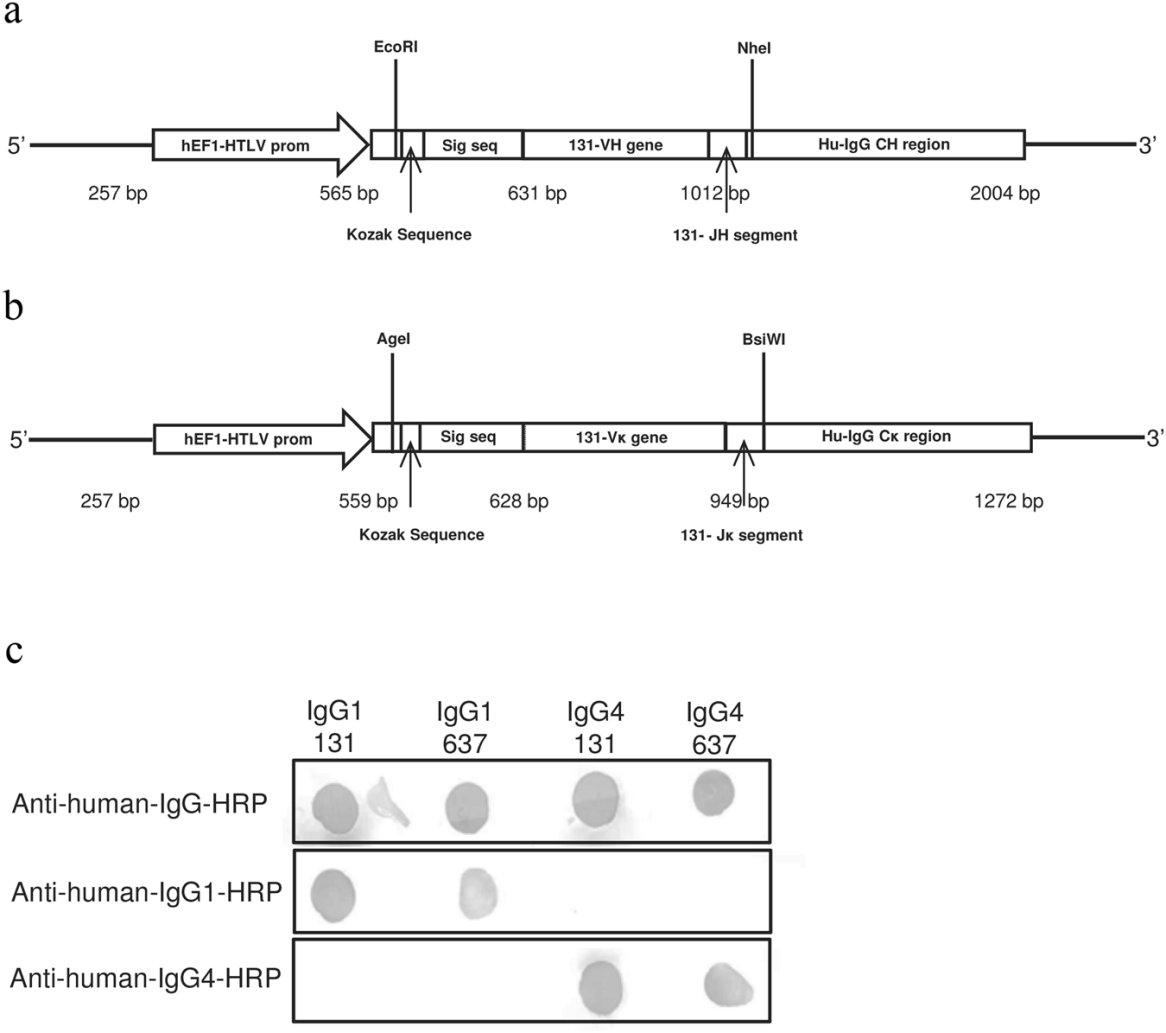
Overview of IgG1-131 & IgG4-131 cloning strategy and production. The expression cassettes for the production of **(a)** VH-CH and **(b)** Vκ-Cκ segments of the mAb 131 in IgG format are shown. **(c)** Isotype specificities of the recombinant antibodies along with control mAbs were analyzed by dot blot using isotype specific HRP-conjugated antibodies.

For recombinant expression of mAb 131, the V regions of heavy and light chains were synthesized *de novo* and cloned into plasmids for the transient expression of IgG1 and IgG4 isotypes in HEK293 cells (see Fig. 2a and b for schematic representations of the constructs for VH-CH and Vκ-Cκ, respectively). The successful secretion of the antibodies into the medium was verified by dot-blot with anti-human IgG, IgG1 and 4 isotype-specific HRP-conjugated secondary antibodies (Fig. 2c).

### IgG1-131 binds the γ-subunit of fetal AChR with high specificity and affinity

**Figure 3:**
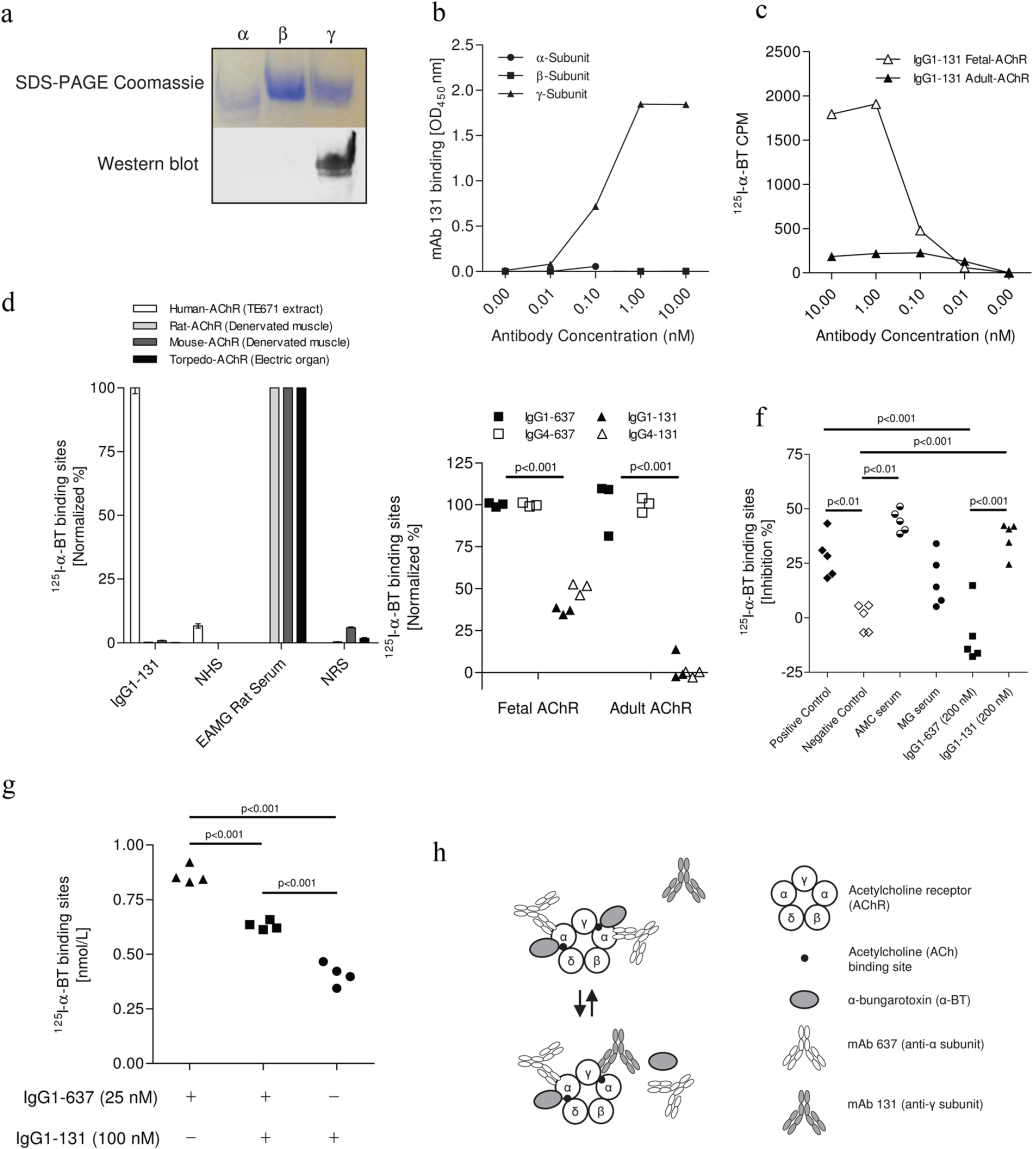
Recombinant IgG1-131 binds fetal AChR and γ-subunit with high specificity and apparent affinity. **(a)** The α, β and γ-subunits of the human muscle AChR were resolved on SDS-PAGE (shown in upper panel) and specificity of IgG1-131 for γ**-**subunit was analyzed by Western blot (lower panel). Full image of the SDS-PAGE and Western blot are shown in Supplementary Fig. S1. **(b)** ELISA analysis of IgG1-131 (0.01 nM – 10 nM) binding to the α, β and γ-subunit of the AChR. **(c)** Radio-immunoprecipitation of IgG1-131 using fetal and adult AChR extracted from TE671 and CN21 cell membranes respectively. **(d)** Cross-reactivity of IgG1-131 against rat and mouse muscle AChR, and the *Torpedo* electric organ AChR was analyzed by radio-immunoprecipitation assay. **(e)** The specificities of IgG1-131 and IgG4-131 were tested against fetal (TE671 membrane extract) and adult AChR (CN21 membrane extract). IgG1-637 and IgG4-637 were used as a reference in both tests. **(f)** ^125^I-α-BT blocking was measured as percentage inhibition in the ^125^I-α-BT binding to AChRs after co-incubating the individual antibody/AMC serum and ^125^I-α-BT labelled AChR. The results are from two independent experiments. **(g)** Analysis of competition between IgG1-131 and IgG1-637. IgG1-131 (100 nM) when co-incubated with IgG1-637 (25 nM), reduced the amount of ^125^I-α-BT co-precipitated with fetal AChR compared to 25 nM IgG1-637 alone. **(h)** Schematic representation of the binding effects of mAb 131 and mAb 637. The mAb 637 binds to the two designated α-subunits of the fetal AChR, allowing simultaneous binding of two α-BT molecules. However, in the presence of competing/blocking mAb 131, one of the α-BT molecules and mAb 637 at the α/γ interface gets displaced by mAb 131.

IgG1-131 specifically recognized the recombinant γ-subunit of the AChR in Western blots (Fig. 3a) of recombinant α, β and γ extracellular domains of the AChR (produced in yeast). The γ-subunit specificity was confirmed by ELISA, where IgG1-131 bound to recombinantly expressed γ-subunit, but not to the α or β subunit (Fig. 3b). By radioimmunoprecipitation using cell extracts containing the complete AChR radiolabeled with ^125^I-α-bungarotoxin (^125^I-α-BT), IgG1-131 precipitated the fetal AChR, but not the adult AChR (Fig. 3c). More than half of the fetal AChR in the assay could be precipitated by 1 nM IgG1-131, suggesting a subnanomolar apparent affinity of the antibody. The recombinant antibody IgG1-131 did not show any cross-reactivity with AChR derived from extracts of rat and mouse denervated muscles or *Torpedo californica* electric organ (Fig. 3d), as previously demonstrated with the original P90-131 B-cell line^20^. In a radioimmunoprecipitation assay, mAb 131 binding to the γ-subunit partially prevented binding of α-BT to the fetal AChR: the ^125^I-α-BT bound to fetal AChR in the presence of IgG1-131 (10 nM) was approximately 60% less when compared to a control mAb IgG1-637 that is specific for the α-subunit of the AChR (p < 0.001, Fig. 3e).

The results in Fig. 3e were consistent with the notion that IgG1-131 displaced ^125^I-α-BT from one of its two binding sites, similar to that seen with AMC serum which displaced ~50% of ^125^I-α-BT from the fetal AChR^26^. To investigate this we used a clinical diagnostic assay to test displacement of ^125^I-α-BT from the AChR. In line with the findings by Riemersma *et al*.^26^, we found that serum from a patient with a history of 4 pregnancies affected by AMC blocked ~50% of ^125^I-α-BT binding to the AChR. IgG1-131 blocked ^125^I-α-BT binding to a similar degree (Fig. 3f). As expected, the negative control and IgG1-637 did not block ^125^I-α-BT binding to the AChR. Accordingly, there was a significant difference between IgG1-131 and the negative control (p < 0.001), and also between IgG1-131 and IgG1-637 (p < 0.001). Since bungarotoxin binds to the α-subunit, while IgG1-131 binds to the γ-subunit, the results also suggested that IgG1-131 binds near the α/γ subunit interface. To see whether this was the case, we performed a competition experiment with IgG1-131 and IgG1-637 in a radioimmunoprecipitation assay: In comparison to IgG1-637 alone, adding an excess of IgG1-131 significantly decreased ^125^I-α-BT-binding to the precipitated AChR (Fig. 3g). Using IgG1-131 alone decreased ^125^I-α-BT-binding to the AChR even further, suggesting that the binding epitopes of both antibodies are too close for simultaneous binding near the α/γ subunit interface, and that only IgG1-131 has a ^125^I-α-BT blocking effect (schematically shown in Fig. 3h).

### Anti-AChR monoclonal antibodies target RMS cell lines

**Figure 4:**
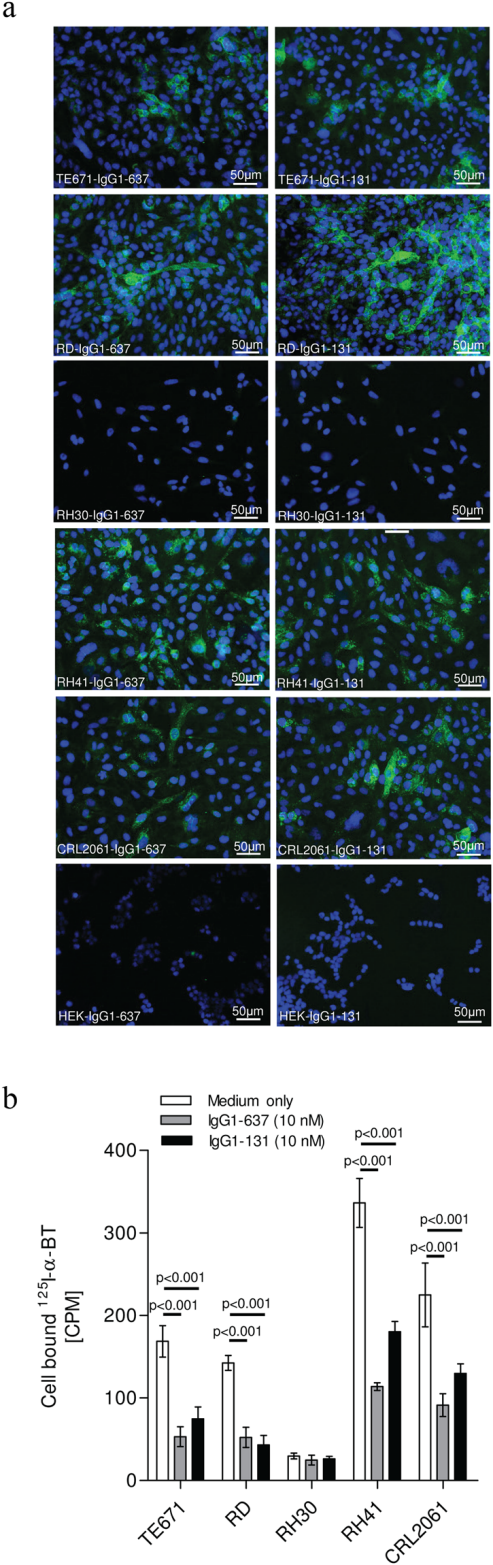
IgG1-131 binds to human RMS cell lines. **(a)** Binding of IgG1-131 and IgG1-637 to cell surface fetal AChR on different RMS cell lines was analyzed by immunofluorescence. Binding of antibody was detected by goat anti-human IgG-Alexa 488 antibody (green) and Hoechst was used to stain the nuclei. HEK293 cells were used as negative control for each antibody. Representative images at 20x magnification are shown, n = 2. Scale bar = 50 µm. **(b)** Analysis of ^125^I-α-BT bound to cell surface AChR on RMS cell lines after incubation with IgG1-131 or IgG1 637. Individual bars correspond to the mean ± SD of three replicates tested for each condition. Results are representative of 3 independent experiments.

Since the fetal AChR is a diagnostic marker of RMS^27^, we were interested to see whether IgG1-131 binding to the 5 well-characterized RMS lines could be detected by immunofluorescence. These included the embryonal lines TE671 and RD, and the alveolar lines RH30, RH41 and CRL2061. As a control for AChR expression, mAb 637 was used in parallel. Immunoreactivity to TE671, RD, RH41 and CRL2061 cells was observed for both AChR antibodies; in contrast, no immunofluorescence was detected in RH30 cells stained by either antibody (Fig. 4a). Moreover, we cultured cells with or without these antibodies and subsequently measured the remaining ^125^I-α-BT binding sites on the cell surface. Binding of ^125^I-α-BT to TE671, RD, RH41 and CRL2061 was observed (Fig. 4b), while binding to RH30 cells was very low. Both AChR antibodies (at a concentration of 10 nM) significantly reduced ^125^I-α-BT binding to TE671, RD, RH41 and CRL2061 cells (p < 0.001), while in RH30, no effect was observed. The low ^125^I-α-BT and IgG binding to RH30 cells suggests lower AChR expression as compared to the other cell lines^17^.

### IgG1-131 does not modulate cell surface AChR

**Figure 5:**
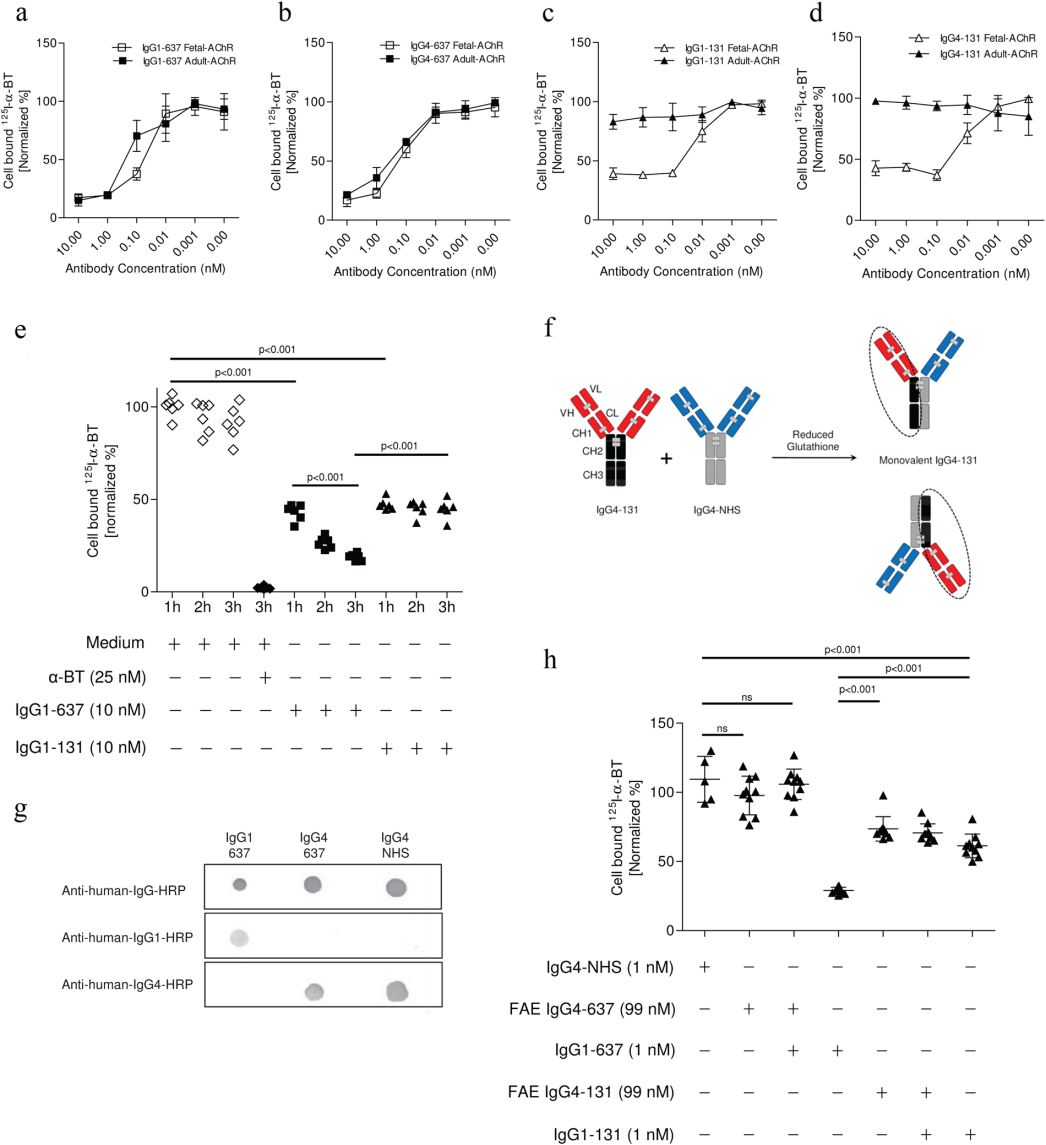
IgG1-131 does not lead to modulation of fetal AChR on TE671 cells, but partly displaces ^125^I-α-BT. The effect of **(a)** IgG1-637, **(b)** IgG4-637, **(c)** IgG1-131 and **(d)** IgG4-131 on the ^125^I-α-BT binding to TE671 (fetal AChR) and CN21 (adult AChR) cells is shown as normalized cell bound radioactivity at varying molar concentrations of the antibodies. Cells were incubated with antibodies for 3 h and subsequently labelled with ^125^I-α-BT. Each data point corresponds to the mean ± SD of three replicates. **(e)** Effect of IgG1-131 and IgG1-637 on ^125^I-α-BT binding to TE671 cells at different time points (1, 2 and 3 h). For each time point, cells cultured in medium without any IgG were used as controls. Unspecific binding of ^125^I-α-BT was measured by pre-incubation of cells with an excess of unlabeled α-BT (25 nM). Data points of 1, 2 and 3 hours incubation were pooled for this condition. **(f)** Schematic representation of the Fab arm-exchange reaction under reducing conditions between IgG4-131 (VH and VL domains shown in red and CH in black color) and IgG4 from normal human serum (IgG4-NHS; VH and VL domains shown in blue and CH in grey color) to yield two bispecific IgG4 molecules. **(g)** Purity of IgG4 isolated from normal human serum (NHS) was validated by dot blot assay along with control mAbs. **(h)** Competition experiments using monovalent FAE IgG4-131 and IgG4-637. The loss of the cell bound radioactivity due to the IgG1-131 incubation was not significantly affected by an excess FAE IgG4-131. In contrast, FAE IgG4-637 completely counteracted the effect of IgG1-637. Data are pooled from 3 experiments with representative groups.

Many AChR antibodies have been shown to cause modulation of cell surface AChRs which is dependent on cross-linking between molecules. Antigenic modulation was initially measured by quantifying the release of ^125^I following the degradation of ^125^I-α-BT-labelled AChRs^28,29^. As explained above for RMS cell lines, we used an alternative method^23,24,30,31^ in which we first incubated the cells with antibody and subsequently with ^125^I-α-BT, to measure the bound ^125^I-α-BT on the surface of the cells. The degradation of AChR can be conveniently detected by reduction of ^125^I-α-BT binding sites, but can be confounded by antibodies that directly block ^125^I-α-BT binding to AChRs^32^. Concentrations of ≥1 nM IgG1-637 or IgG4-637 (unmodified mAb/ not Fab-arm exchanged) both reduced α-BT binding well below 20% of the levels on the cell surface of untreated TE671 and CN21 cells (Fig. 5a/5b) within 3 h  at 37 °C, in line with the strong antigenic modulation capacity of this antibody^33^. However, at a concentration of 0.1 nM or higher, IgG1-131 and IgG4-131 both caused ~50% reduction in binding of ^125^I-α-BT to the TE671 RMS cell (Fig. 5c/5d), in agreement with its ability to block one of the two α-BT binding sites. IgG1-131 had no effect on α-BT binding to the adult AChR (expressed on the CN21 cells), confirming its γ-subunit specificity. Already after 1 h of incubation, both IgG1-131 and IgG1-637 (at 10 nM) reduced α-BT binding to the surface of TE671 by 50% (Fig. 5e). The reduction by IgG1-131 is thought to derive from displacement of one of the two ^125^I-α-BT from the AChR. During the following 2 hours, α-BT binding continued to decrease significantly in IgG1-637-treated cells. In contrast, during this time period, α-BT binding remained stable in IgG1-131-treated cells, suggesting that it did not induce antigenic modulation.

To further study a potential modulation effect of mAb 131, we generated a monovalent IgG4 version of the antibody by Fab arm-exchange (FAE), as described^33,34^. During FAE, mono-specific IgG4 antibodies randomly exchange “half-molecules” (consisting of a heavy chain – light chain pair) giving rise to bispecific IgG4 molecules (Fig. 5f) that lose the ability to cross-link the same antigen and can be considered as functionally monovalent. To set up an *in vitro* FAE reaction, IgG4 was purified from normal human serum and validated by a dot blot assay along with IgG1-637 and IgG4-637 (Fig. 5g). As expected, the modulatory effect of control antibody IgG1-637 (which does not block ^125^I-α-BT binding) was completely neutralized by a 99 fold excess of FAE IgG4-637 antibody (p < 0.001, Fig. 5h). Similar to IgG1-131, Fab-arm exchanged IgG4-131 strongly reduced ^125^I-α-BT binding to TE671 cells (Fig. 5h). A 99-fold molar excess of Fab-arm exchanged IgG4-131 antibody did not significantly counteract the reduction of ^125^I-α-BT binding sites on TE671 cells caused by 1 nM IgG1-131. This suggests that the antigenic modulation capacity of IgG1-131 is absent or very low and that instead it mainly reduced ^125^I-α-BT binding to the fetal AChR.

### IgG1-131 does not inhibit fetal AChR currents in transfected HEK293 cells

**Figure 6:**
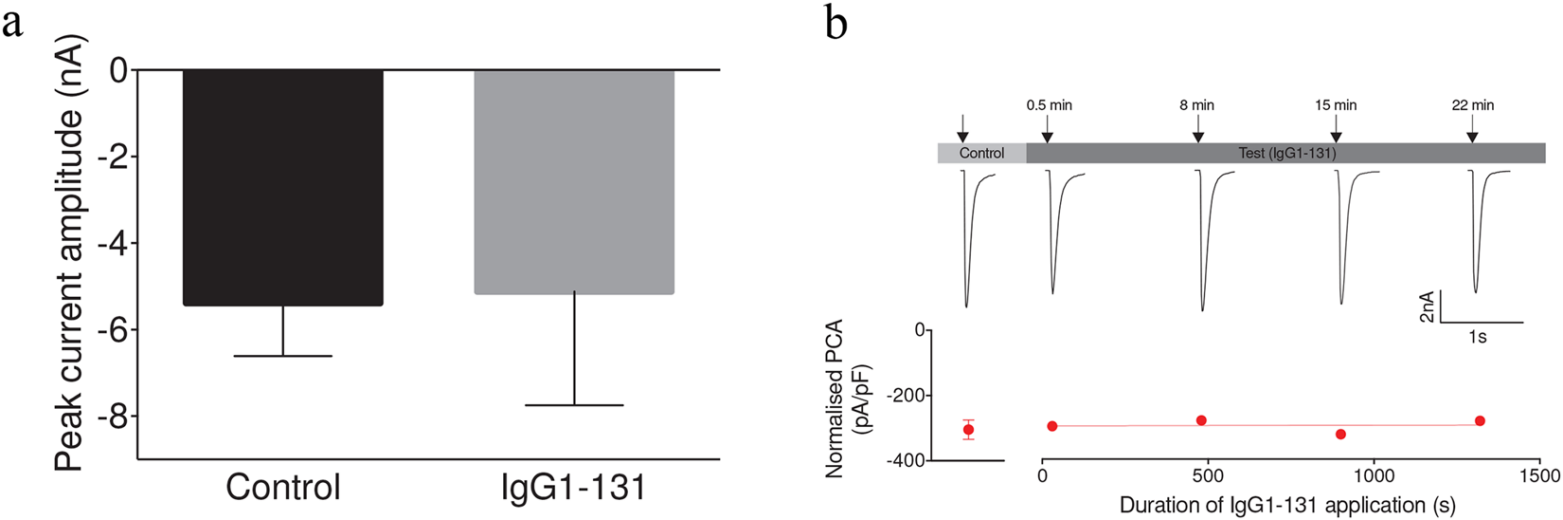
IgG1-131 does not inhibit fetal AChR function. **(a)** AChR currents (mean ± SEM) in individual cells (n = 6) were measured during the continuous perfusion with control solution (without IgG1-131) and with test solution (application of 5 μg/mL purified IgG1-131 for 2.5 min). **(b)** Repetitive AChR current measurements in an individual cell continuously perfused with 10 mL of test solution. Data points are the means ( ± SEM) of a number of measurements (control solution: n = 10, test solution: n = 6 at 0.5 min, n = 6 at 8 min, n = 6 at 15 min and n = 3 at 22 min). AChR currents were normalized to the cell capacitance and expressed as pA/pF.

As IgG1-131 partially blocked binding of α-BT, which is well known to compete with ACh and other agonists or competitive antagonists for the ACh binding site, we asked whether IgG1-131 itself inhibited AChR function. To this end, whole-cell currents were measured in transfected HEK293 cells expressing fetal AChR in the presence or absence of IgG1-131. ACh applications of 1 s duration were performed to elicit AChR currents. All recordings were started with control solutions lacking IgG1-131 (“control solution”) followed by the “test solution” containing 33 nM IgG1-131. Compared to control solution there was no current reduction during a 2.5 min application of the test solution (peak current amplitude of −5.4 ± 1.2 nA and −5.1 ± 1.1 nA, respectively; p = 0.87; Fig. 6a). Even continuous perfusion with IgG1-131 containing test solution for more than 20 min did not result in any significant current reduction (Fig. 6b), suggesting that mAb131 did not block AChR function.

## Discussion

The fetal AChR is expressed mainly during development and has relevance for human disease. In a rare form of AMC, fetal-specific inhibitory antibodies from the mother cause fetal paralysis and often fetal death during gestation. In RMS, the fetal AChR is expressed on the cell surface and may provide a target for specific immunotoxins. In this study the patient-derived recombinant IgG1-131, cloned by B cell immortalization from the thymus of an MG patient, was shown to bind to the fetal AChR with high apparent affinity, to prevent binding of α-BT to one of its two sites on the fetal AChR, but not to cause internalization or inhibit the ion channel-function of the fetal AChR.

Since mAb 131 was isolated by B cell immortalization, this specificity is associated with the original heavy and light chain partners. The antibody V genes had accumulated a large number of overt mutations in the CDRs. The high replacement to silent (R/S) mutation ratio among V genes indicated that the somatic hypermutation of the antibody was driven by its antigen^35,36^.

Our results also showed that mAb 131 has only a weak capacity for antigen modulation and merely displaces one of the ^125^I-α-BT molecules. Apparently, in our previous study^20^, we had incorrectly interpreted the loss of cell bound α-BT induced by mAb 131 as due to antigen modulation.

IgG1-131 bound specifically to the γ-subunit of the fetal AChR, but partially competed with α-subunit-specific IgG1-637. Since IgG1-131 did not affect the ACh-induced receptor currents, the results suggest that the antibody binding site is located on the α/γ interface and overlaps with the α-BT binding site, but not with the binding site for ACh. Previous reports showed that the sera of healthy mothers with obstetric history of AMC^7,26^ and sera of 5 out of 12 parous women with MG^7^ contained antibodies that inhibited α-BT binding to the fetal AChR by ~50% and also blocked the fetal AChR response to ACh in electrophysiological and^22^Na flux studies. However, the administration of α-BT blocking anti-AChR antibodies purified from MG serum has also been reported to protect mice from *in vivo* toxicity of α-BT without inducing any of the clinical MG symptoms in the animals^37^. In another study, the α-BT blocking antibody titers correlated less with the changes in muscle weakness when compared to the binding antibody titers^38^. These observations suggest additional antigenic epitopes on the fetal specific gamma subunit, and at the alpha/gamma interface; antibodies binding to these epitopes do not have pathogenic effects but could protect against pathogenic antibodies.

Since α-BT has a size of ~8000 Da, in comparison with 162 Da for ACh, it is perhaps not surprising that antibodies, when binding to various subunits of the AChR, more commonly block α-BT rather than ACh binding and that these two blocking effects do not always coincide. So far, monoclonal antibodies generated using thymic B cells from MG patients^7,22,24^, including some that are specific for the fetal AChR have not been reported to interfere with AChR function, but the example of mAb 131 and several other antibodies^7^ confirms that the fetal AChR is a major target of B cells in the thymus of EOMG patients.

While it is clear that mAb 131 is not pathogenic by antigenic modulation, it could, being of the IgG1 isotype, still damage the fotus by activation of complement. It is known that the early gestation stage (12 weeks) is marked by the maturation of the neuromuscular junction in the masseters (and presumably other muscles) of the human fetus^39^ and, therefore, overactivation of the complement system, associated with normal pregnancies^40–42^ might potentially contribute to a pathogenic effect of mAb 131. In other antibody-mediated diseases, such as antiphospholipid syndrome (APS) and systemic lupus erythematosus (SLE), the complement system is involved in transfer of the disease from mother to child: a pathogenic effect of APS antibodies due to complement activation has been shown in a mouse model and in human placental tissue of SLE and APS subjects^43^.

A ‘non-pathogenic’ role of mAb 131 could be of interest for RMS diagnosis and therapy^15,17^, because this cancer type expresses high amounts of AChRs of the fetal type. Indeed, mAb 131 was found to bind specifically, in the nanomolar range, to various human RMS cell lines of embryonal and alveolar origin as seen with immunofluorescent staining. This suggests that mAb 131 could be of diagnostic or clinical relevance and encourages further development for use of this antibody to target RMS.

## Methods

### Sequence analysis

The variable heavy and light chain genes of mAb 131 were PCR amplified with VH/JH and Vκ/Jκ primer and sequenced^20^. The sequences were analyzed by comparison with human germline alleles using the IMGT/V-QUEST program (http://www.imgt.org/IMGT_vquest/share/textes/).

### Cloning and expression of fully human IgG1-131 and IgG4-131

The VH and Vκ expression cassettes were designed by the incorporation of the restriction sites (5′*EcoRI*/3′*NheI* for VH and 5′*AgeI*/3′*BsiWI* for Vκ respectively) compatible with the pFUSE expression system (Invivogen/Life Technologies), Kozak translation initiation sequence, and a signal peptide from pIgG1-637^33^; both constructs were chemically synthesized by GeneArt. The VH and Vκ genes were sub-cloned respectively into the pFUSE-CHIg-hG1, pFUSE-CHIg-hG4 and pFUSE-CLIg-hκ vectors (Fig. 2a/b). HEK293 cells were cultured in DMEM (Gibco, Cat No. 10938-025) supplemented with 50 U/mL penicillin, 50 U/mL streptomycin & 1% L-glutamine and co-transfected with heavy and light chain plasmids (1 μg:1.5 μg/10^6^ cells) using 1:3 ratio of DNA and linear polyethylenimine (PEI, Polyscience Inc, Cat No. 23966). An equivalent amount of medium with 10% FCS was added to the cultures 4 h after the transfection. The cells were cultured for 5 to 7 days. To verify successful expression, the secreted IgG in the supernatant was concentrated by Amicon Ultra-15 centrifugal filter units (MWCO of 100 kDa, Millipore, Cat No. UFC910024) and measured with a sandwich ELISA using goat F(ab’)_2_ anti-human IgG-Fcγ (Jackson Immuno-Research, Cat No. 109-006-008; diluted 1:200) for coating, and bound IgG was detected with HRP-conjugated goat F(ab’)_2_ anti-human IgG-Fcγ (Jackson Immuno-Research, Cat No. 109-036-008; diluted 1:20,000). The concentration of the expressed antibody was calculated relative to the human serum IgG standards as μg/mL.

IgG was purified from 50 mL culture supernatant using a HiTrap Protein G HP column (GE Healthcare Life Science, Cat No. 17-0404-01) in an AKTA explorer 900 system (GE Healthcare Life Science), eluted with 0.2 M sodium citrate pH 3.0, and concentrated using a Centricon Plus-20 centrifugal filter device (MWCO of 30 kDa, Millipore, Cat No. UFC2 LTK 08). Purified mAbs IgG1-131, IgG4-131, as well as mAbs IgG1-637 & IgG4-637 as controls, were blotted on nitrocellulose membranes (Biorad, Cat No. 162-0116) for 1 h at 37 °C, blocked with 5% non-fat dry milk in 1x PBS for 1 h at 37 °C, washed with PBS-T (containing 0.05% Tween20) and incubated with HRP-conjugated goat F(ab’)_2_ anti-human IgG-Fcγ (Jackson Immuno-Research, Cat No. 109-036-008; diluted 1:2500) or mouse anti-human IgG1 (Invitrogen, Cat No. 05-3320; diluted 1:2500) or mouse anti-human IgG4 (AbD serotec, Cat No. MCA2098P; diluted 1:2500) secondary antibody. The blots were developed with 20% DAB in sodium acetate citric acid buffer pH 5.5 activated by 5 μL 30% H_2_O_2_.

### Analysis of antibody binding to AChRs

The binding of antibodies to cell membrane extracts containing the fetal AChR (expressed on the TE671 rhabdomyosarcoma cells) and the adult AChR (expressed on the CN21 cells) was detected in a standard radio-immunoprecipitation assay. 10 nM each of IgG1-131 and IgG1-637 were incubated overnight at 4 °C with respective cell extracts (~3 fM of human AChR) labelled with an excess of ^125^I-α-BT (3.4 TBq/mM, Perkin Elmer, Cat No. NEX126) and normal human serum as a co-precipitant/carrier. The immune-complexes were precipitated by adding 150 μL of goat serum with anti-human IgG at 4 °C and gamma counts were recorded after 4 h. Results were expressed as normalized values or as co-precipitated ^125^I-α-BT binding sites per liter of sample in nmol/L.

IgG1-131 and α-BT competition was also tested using ARAb blocking RIA following the manufacturer’s instructions (IBL International, Cat No. RE21033). The assay is based on competitive binding of anti-AChR antibodies in a sample and ^125^I-α-BT to the AChR. Results were expressed as percentage inhibition of ^125^I-α-BT binding to AChRs.

The binding of IgG1-131 to AChR in membrane extracts from rat denervated muscle (100 μL), mouse denervated muscle (50 μL) and *Torpedo* electric organ (0.05 μg) was determined by radio-immunoprecipitation as described above. Normal human and rat sera were included as negative controls while EAMG (experimental autoimmune myasthenia gravis) rat serum was included as a positive control for murine and *Torpedo* AChR detection.

The specificity of IgG1-131 was further determined by Western blot analysis using 5 μg recombinantly expressed extracellular domains (ECD) of the human AChR subunits from the yeast *Pichia pastoris* ^44^, that were resolved on 10% SDS-PAGE under reducing condition and transferred to a PVDF membrane (Millipore, Cat No. IPFL00010). The blotted subunits were blocked with 1:2 dilution of Odyssey^TM^ blocking buffer (LI-COR, Cat No. P/N 927-40100), incubated with 1:100 dilution of IgG1-131 for 1 h and then incubated with IRDye 800CW goat anti-human IgG (H + L) (LI-COR, Cat No. 926-32232) for 1 h at a dilution of 1:5000. The membrane was scanned using the Odyssey CLx imaging system. Additionally, the affinity of IgG1-131 against the fetal AChR and recombinant subunits of human muscle AChR was determined at varying antibody concentrations (0.01 nM - 10 nM) by radio-immunoprecipitation assay and an indirect antigen ELISA, respectively^23^.

### AChR staining on RMS cell lines

RMS cell lines were cultured on poly-D-lysine-coated (1%) glass coverslips (12 mm diameter, n = 6) in a six well tissue culture plate up to 75% confluence. Thereafter, medium was removed; cells were washed once with PBS, fixed using 3% paraformaldehyde solution for 5 min, washed once with PBS and blocked with 5% goat serum for 30 min. The coverslips were then removed from the culture plate and incubated upside-down on a 50 μL drop of PBS with 3 μg/mL of IgG1-131, IgG1-637, normal human serum IgG or 1% BSA along with 2 μg/mL Hoechst stain (Sigma Aldrich, Cat No. B2261) for 1 h. The coverslips were washed sequentially three times by dipping in 1x PBS followed by incubation on 50 μL drops of 1:1000 diluted goat anti-human IgG-Alexa 488 antibody (Molecular Probes/Invitrogen, Cat No. A11013). Coverslips were mounted on gelatin coated glass slides using 10 μL of 80% glycerol in PBS and analyzed at 20x magnification on an Olympus BX51 fluorescence microscope.

### *In vitro* cell surface ^125^I-α-BT binding and AChR antigenic modulation assay

RMS cell lines of alveolar origin (RH30, RH41 & CRL2061) were cultured in RPMI 1640 (Gibco, Cat No. 12633-012) with 10% FCS and embryonal RMS cell lines (TE 671,CN21 & RD) were maintained in DMEM (Gibco, Cat No. 10938-025) supplemented with 10% FCS. Other supplements included 50 U/mL penicillin and streptomycin, 1% L-glutamine, 1% sodium pyruvate and 0.1% dexamethasone. The cells were seeded into a 48 well tissue culture plate and grown to confluence for 4 days. The confluent cultures were incubated with different molar concentrations (0.001 nM–10 nM) of test and control mAbs diluted in an incubation medium (DMEM supplemented with 40 μM cycloheximide) for 1–3 h at 37 °C/5% CO_2_. Cells were washed once with 1x PBS and further incubated with the labeling medium (100 μL DMEM containing 1:5000 ^125^I-α-BT) for 1 h at 37 °C/5% CO_2_. The cells were washed three times with 1x PBS and then lysed by 0.5 N NaOH for 10 min before measuring ^125^I gamma counts. The non-specific binding of ^125^I-α-BT to the cells was measured as gamma counts in samples that had been pre-incubated with 25 nM unlabeled α-BT. The residual cell bound radioactivity (^125^I-α-BT) was normalized to the ^125^I-α-BT CPM of the cells incubated with the corresponding culture medium only.

### *In vitro* Fab arm-exchange and cell surface fetal AChR protection

IgG4 from 1:2 diluted normal human serum was purified by AKTA explorer 900 through a CaptureSelect human IgG4 affinity matrix (Life Technologies, Cat No. 290005) and analyzed by dot blot as described in “Cloning and expression of full IgGs” section (Fig. 5g). *In vitro* FAE was induced by co-incubating 10 μg/mL IgG4-131 or IgG4-637 with 500 μg/mL IgG4 (1:50) purified from normal human serum with 0.5 mM reduced glutathione (Merck, Cat No. 1040900005) at 37 °C for 24 h^34,45^. The samples from the FAE reactions were desalted using Amicon Ultra-0.5 centrifugal filter units (MWCO 30 kDa, Millipore, Cat No. UFC503024). Exchanged IgG4 were used to test competition with unmodified IgG1-131 or 637 in antigenic modulation experiments with TE671 cells. Exchanged (monovalent) IgG4 samples contained a 99 fold excess molar concentration of AChR-binding antibody (measured by radio-immunoprecipitation) compared to unmodified (cross-linking) AChR antibodies. Relevant controls in the form of normal human IgG4 and only Fab arm-exchanged IgG4 were included.

### Cell surface fetal AChR functional assay

Patch clamp experiments were performed on HEK293 cells transiently transfected with fetal AChR subunits and EGFP using PEI, with cells at ~70% confluence one day after plating in six well tissue culture dishes. Briefly, cDNA encoding the human muscle AChR α, β, δ, and γ-subunits were cloned into the mammalian expression vector pcDNA3.1 (Invitrogen). These were co-transfected with pEGFP-N1 (Invitrogen), which contains cDNA encoding EGFP. After 5–7 h of exposure to a mix of 3 μg cDNA, 20% glucose and PEI in 2 mL of culture medium, the cells were plated onto poly-L-lysine-coated coverslips, and patch-clamped between two and three days later.

All experiments were performed at room temperature using the whole-cell patch clamp configuration. Recording pipettes were made of thin-walled borosilicate glass (GC150TF-10, Harvard Apparatus). Pipette tips were fire-polished to a final resistance of 2–4 MΩ (MF-900 Microforge, Narishige). Extracellular solutions contained (in mM): NaCl 150, KCl 2.8, HEPES 10, MgCl_2_ 2, CaCl_2_ 2 and glucose 10 with pH adjusted to 7.4 using NaOH. Pipette solutions contained (in mM): NaCl 4, KCl 144, HEPES 10, MgCl_2_ 2, ATP 2 and EGTA 10 with pH adjusted to 7.2 using KOH. All measurements were performed at a holding potential of −60mV. Currents were amplified using an Axopatch-1D amplifier (Molecular Devices) and, after filtering at 5 kHz, sampled to hard disk at 25 kHz. Data analysis was performed with pClamp 10.5 (Molecular Devices).

Fast solution exchange was accomplished using the modified HSSE-2/3 application system (ALA Scientific Instruments). A four-barrel perfusion pipette with a tip diameter of around 300 μm was used to switch between control and test solutions, both of which consisted of an agonist containing solution for receptor stimulation (extracellular solution with 1 mM acetylcholine) and an agonist-free solution for agonist removal (extracellular solution). In contrast to the control solution, the test solution additionally contained 5 μg/mL purified IgG1-131 in both the agonist containing and the agonist-free solution.

### Statistical analysis

Statistical analysis was done using Prism software version 5.00 (GraphPad). Binding of IgG1-131 and IgG1-637 to fetal and adult AChR extracts and RMS cell modulation was analyzed by a two way ANOVA with Bonferroni posttest. Data from IgG-131/IgG-637 competition, the AChR blocking RIA and antigenic modulation of Fab arm-exchanged samples were analyzed by one-way ANOVA and Bonferroni multiple comparison test. AChR currents in individual cells were analyzed by t-test, while repetitive AChR current measurements were analyzed by regression analysis. P values equal to or less than 0.05 were considered as significant.

## Electronic supplementary material

Supplementary Information
