# Supplementary material for: Characterization of an anti-fetal AChR monoclonal antibody isolated from a myasthenia gravis patient
Source: Sci Rep. 2017 Oct 31;7:14426. doi: 10.1038/s41598-017-14350-8 (PMC5663942; doi:10.1038/s41598-017-14350-8)

Supplementary information:

Characterization of an anti-fetal AChR monoclonal antibody isolated from a myasthenia gravis patient

Abhishek Saxena, Jo Stevens, Hakan Cetin, Inga Koneczny, Richard Webster, Konstantinos Lazaridis, Socrates Tzartos, Kathleen Vrolix, Gisela Nogales-Gadea, Barbie Machiels, Peter C. Molenaar, Jan Damoiseaux, Marc H. De Baets, Katja Simon-Keller, Alexander Marx, Angela Vincent, Mario Losen and Pilar Martinez-Martinez

a

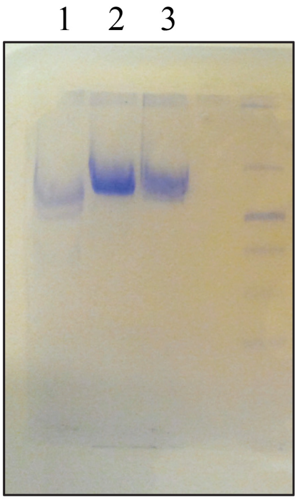

b

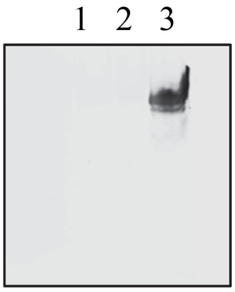

1 – AChR ECD α-subunit
2 – AChR ECD β-subunit
3 – AChR ECD γ-subunit

Supplementary Figure S1. Immune reactivity of IgG1-131 to AChR γ-subunit. (a) The recombinant extracellular domains of human AChR subunits (a, β and γ) were resolved on 10% SDS-PAGE under reducing condition and stained by Coomassie blue. (b) Western blot depicting the immune reactivity of IgG1-131 to the recombinant extracellular γ-subunit of the human AChR.

SDS-PAGE gel at different exposures

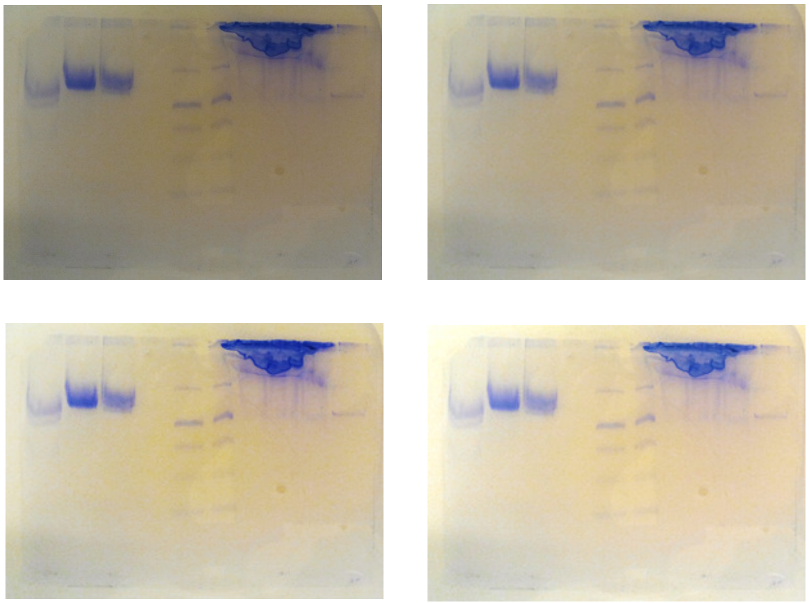

Western blot at different exposures

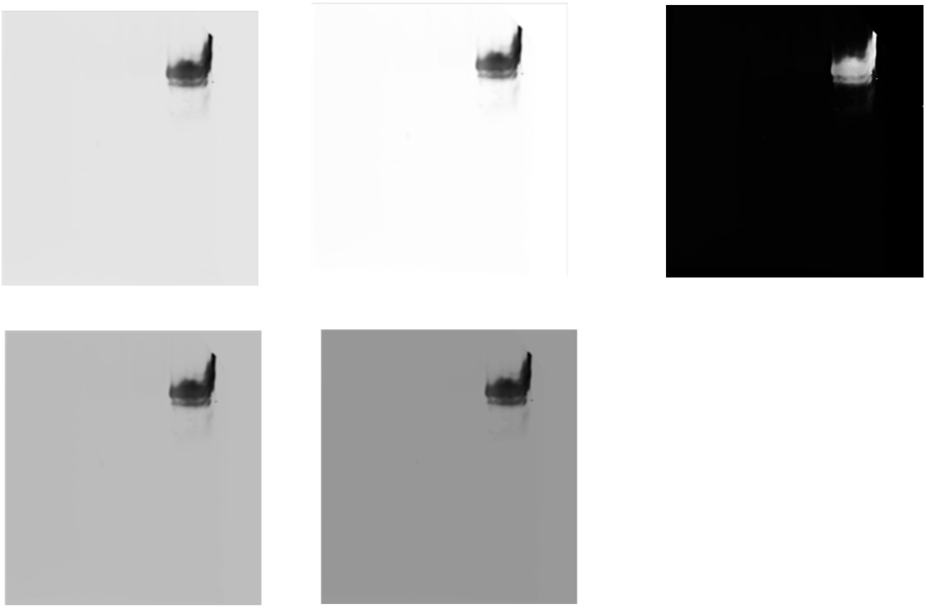

Supplement: Supplementary file 1 — Supplementary Information [file 41598_2017_14350_MOESM1_ESM.pdf]
